# Inflammation and delirium in critically Ill patients: a review

**DOI:** 10.3389/fnagi.2026.1783455

**Published:** 2026-09-03

**Authors:** Liang Li, Xiaoli Wang, Jialin Liu

**Affiliations:** 1 Department of Geriatrics, Ruijin Hospital, Shanghai Jiao Tong University School of Medicine, Shanghai, China; 2 Department of Critical Care Medicine, Ruijin Hospital, Shanghai Jiao Tong University School of Medicine, Shanghai, China; 3 College of Health Science and Technology, Shanghai Jiao Tong University School of Medicine, Shanghai, China

**Keywords:** anti-inflammatory therapy, biomarkers, critically ill patients, delirium, inflammation, inflammatory biomarkers, neuroinflammation, treatment

## Abstract

The high prevalence and adverse impact of delirium in critically ill patients necessitate a deeper understanding of its mechanisms. This review explores the pivotal interplay between systemic inflammation and delirium, examining how insults like age, pain and surgery orchestrate neuroinflammation and delirium through microglial activation and blood-brain barrier disruption. Our synthesis identifies a spectrum of inflammatory and neuronal injury biomarkers with prognostic value and evaluates the burgeoning field of anti-inflammatory pharmacotherapy. We conclude that bridging this mechanistic knowledge to clinical practice via biomarker-driven trials is essential for developing effective interventions against delirium.

## Introduction

Critical illness refers to an acute, life-threatening condition characterized by actual or impending organ dysfunction requiring intensive monitoring and, frequently, organ-supportive therapy (Singer et al., 2016; Marshall et al., 2017). In clinical practice, critically ill patients are commonly identified by admission to an intensive care unit, the need for invasive or non-invasive mechanical ventilation, vasopressor or inotropic support, renal replacement therapy, or the presence of severe sepsis, shock, acute respiratory failure or multiple organ dysfunction. Severity of illness is commonly assessed using validated scoring systems such as the Acute Physiology and Chronic Health Evaluation II, the Sequential Organ Failure Assessment score and the Simplified Acute Physiology Score II (Knaus et al., 1985; Vincent et al., 1996; Le Gall et al., 1984).

Delirium is an acute and fluctuating disturbance in attention, awareness and cognition that develops over a short period and is attributable to an underlying medical condition, intoxication or withdrawal, medication exposure or multiple interacting causes (American Psychiatric Association, 2013; Wilson et al., 2020). In critically ill patients, delirium is commonly assessed using validated bedside instruments such as the Confusion Assessment Method for the ICU and the Intensive Care Delirium Screening Checklist (Ely et al., 2001; Bergeron et al., 2001). Delirium may be classified according to motor phenotype as hyperactive, hypoactive or mixed, and according to clinical context as ICU-acquired delirium, sepsis-associated delirium, postoperative delirium, withdrawal-related delirium or delirium superimposed on dementia (Peterson et al., 2006; Slooter et al., 2020).

Delirium is particularly common in critically ill patients, affecting approximately 30–50% of general ICU patients and up to 60–80% of mechanically ventilated patients, depending on illness severity, diagnostic approach and patient population (Ely et al., 2001; McNicoll et al., 2003; Salluh et al., 2015; Sakusic and Rabinstein, 2025). ICU delirium is associated with increased mortality, prolonged mechanical ventilation, longer ICU and hospital stay, greater health-care costs and persistent cognitive and functional impairment after discharge (Girard et al., 2010; Pandharipande et al., 2013; Lobo-Valbuena et al., 2023). Longer delirium duration is also associated with worse long-term cognitive and executive function (Pandharipande et al., 2013; Wolters et al., 2017).

The high burden of delirium in critical illness reflects the distinctive physiological environment of the ICU. Patients are frequently exposed to systemic inflammation, hypoxaemia, shock, cerebral hypoperfusion, metabolic derangements, sedative and analgesic medications, sleep fragmentation, pain, immobilization and organ dysfunction (Devlin et al., 2018; Stollings et al., 2021). These insults may converge on the brain through blood–brain barrier disruption, endothelial dysfunction, neurotransmitter imbalance, mitochondrial injury, impaired synaptic signaling, altered cerebral connectivity and neuroinflammation (Cunningham and Maclullich, 2013; Maldonado, 2013). Advanced age, frailty, dementia and multimorbidity further reduce cerebral reserve and increase vulnerability to acute brain dysfunction (Inouye et al., 2014; Bellelli et al., 2024).

This Review examines inflammation-related mechanisms of delirium in critically ill adults. We focus on systemic inflammatory states relevant to ICU-level illness, including sepsis, major surgery requiring intensive monitoring, pain, aging and frailty (Cunningham and Maclullich, 2013; Maldonado, 2013; Inouye et al., 2014). Evidence from postoperative delirium is included where surgical stress overlaps with critical illness or provides mechanistic insight relevant to ICU delirium (Evered et al., 2018; Berger et al., 2023). We do not aim to cover all forms of delirium comprehensively. Instead, we evaluate how inflammation interacts with broader pathophysiological processes in critical illness and assess whether inflammatory biomarkers and anti-inflammatory therapeutic strategies can inform delirium prediction, prevention and treatment (Brummel et al., 2024; Mosharaf et al., 2025).

This article is a narrative review that summarizes and critically discusses current evidence regarding the relationship between systemic inflammation and delirium in critically ill patients. Relevant literature was identified through searches of major biomedical databases, including PubMed/MEDLINE, Web of Science, Embase, and Google Scholar, using combinations of keywords such as “delirium,” “ICU delirium,” “critical illness,” “sepsis-associated encephalopathy,” “postoperative delirium,” “systemic inflammation,” “neuroinflammation,” “cytokines,” “blood-brain barrier,” “microglia,” “biomarkers,” “IL-6,” “CRP,” “S100β,” “GFAP,” “tau,” “neurofilament light chain,” and “anti-inflammatory therapy.” Additional relevant articles were identified from the reference lists of selected publications. This review was designed to provide a broad narrative synthesis of mechanistic, biomarker, and therapeutic evidence rather than a formal systematic review.

## Why critically Ill patients are uniquely vulnerable to delirium

Critical illness creates a biological state in which multiple homeostatic systems fail simultaneously (Singer et al., 2016; Stollings et al., 2021). Systemic inflammation from sepsis, trauma or surgery may alter endothelial function and increase blood–brain barrier permeability, allowing peripheral immune mediators to influence central nervous system function (Cunningham and Maclullich, 2013; Maldonado, 2013). Respiratory failure and shock can impair oxygen delivery and cerebral perfusion, whereas renal and hepatic dysfunction may alter the metabolism and clearance of neuroactive drugs (Devlin et al., 2018; Hughes et al., 2020). Sedatives, opioids and anticholinergic medications further perturb neurotransmitter systems, including cholinergic, dopaminergic, GABAergic and glutamatergic signaling (Hshieh et al., 2018; Slooter et al., 2020). Environmental exposures in the ICU, such as sleep deprivation, noise, continuous lighting and social isolation, disrupt circadian regulation and cognitive orientation (Simons et al., 2018; Hshieh et al., 2019). These factors interact with baseline vulnerability, including advanced age, frailty, dementia and multimorbidity, thereby lowering the threshold for delirium (Inouye et al., 2014; Bellelli et al., 2024).

### Pathophysiological mechanisms of inflammation and delirium

At present, numerous hypotheses suggest that neuroinflammation serves as the underlying mechanism for the onset of delirium (Xin et al., 2023). The dysfunction of neuronal mitochondria, synaptic impairment, and neuronal apoptosis induced by neuroinflammation are considered important mechanisms contributing to delirium (van den Boogaard et al., 2012). This paper centers on inflammation and explores its association with delirium from multiple perspectives, including surgical trauma, infection, aging, and frailty.

## Infection-driven neuroinflammation in ICU delirium: from systemic inflammation to microglial–C1q-mediated synaptic injury

Previous studies have established that infection is a significant risk factor for ICU delirium. Severe systemic infections such as sepsis may trigger brain dysfunction through inflammatory pathway (Smith et al., 2024). The systemic inflammatory effect of sepsis is characterized by the disruption of the pro-inflammatory/anti-inflammatory balance: pathogen-associated molecular patterns such as bacterial lipopolysaccharide (LPS) activate Toll-like receptors, triggering the release of cytokines like IL-1β, IL-6, IL-8, and TNF-α by macrophages and endothelial cells. These inflammatory mediators activate microglia in the brain, initiating a cascade of neuroinflammation, ultimately leading to mitochondrial dysfunction and impaired synaptic transmission in neurons (Stollings et al., 2021). A study has shown that in patients with sepsis, microglia in the hippocampus and C1q complement activation cause damage to neurons and synapses, thereby leading to neurocognitive disorders including delirium. CSF1-R inhibitors, targeting microglia, reduce C1q levels and the number of C1q-labeled synapses, prevent neuronal damage and synapse loss, and improve neurocognitive outcomes (Chung et al., 2023).

## Surgery-induced inflammatory cascades

**FIGURE 1:**
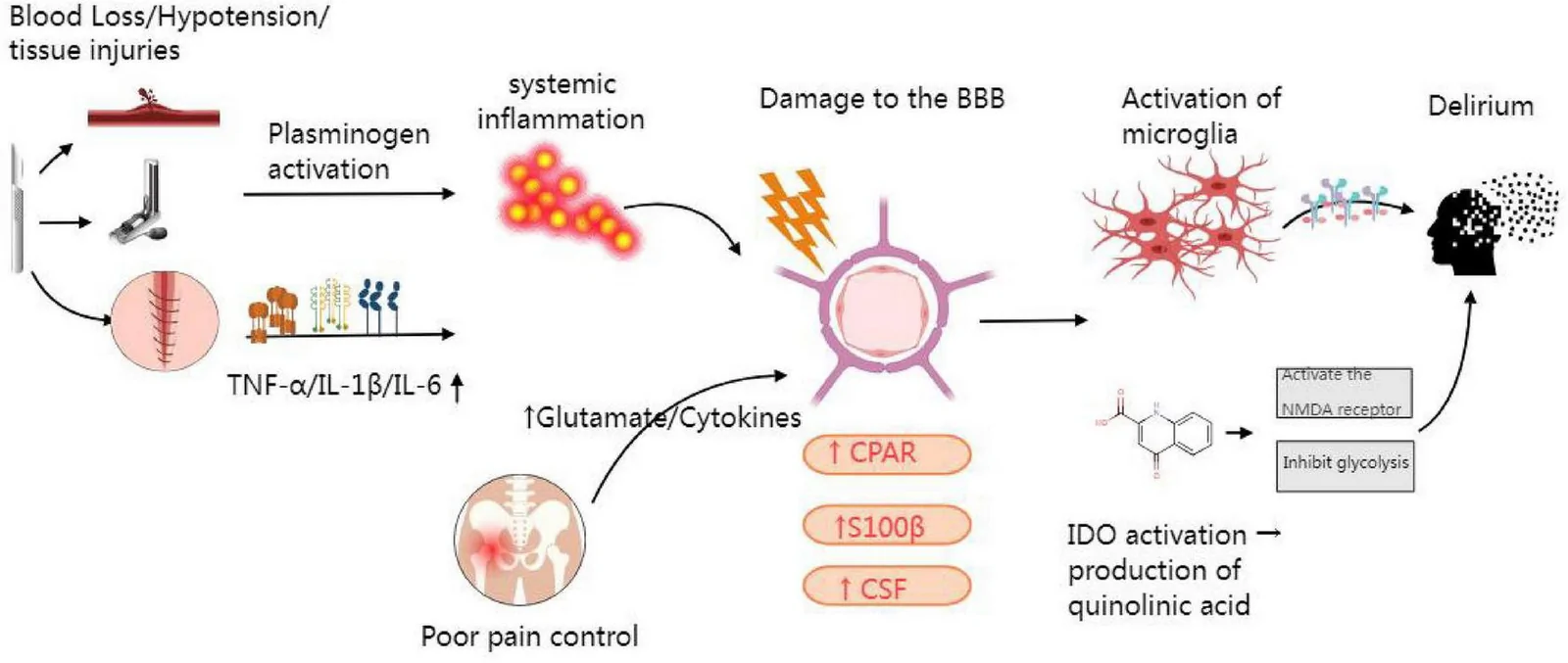
Surgical trauma and delirium. Solid arrows indicate mechanisms supported by clinical or experimental evidence. Black arrows represent direct pathophysiological pathways (e.g., inflammatory signaling, cellular injury), whereas red arrows denote inhibitory or deleterious processes (e.g., blood–brain barrier disruption, neurotoxic effects). Arrow width corresponds to relative pathway contribution. BBB, blood–brain barrier; CNS, central nervous system; CRP, C-reactive protein; DAMPs, damage-associated molecular patterns; EV, extracellular vesicle; GFAP, glial fibrillary acidic protein; HMGB1, high-mobility group box 1; IL, interleukin; NfL, neurofilament light chain; NLRP3, nucleotide-binding oligomerization domain-like receptor family pyrin domain-containing 3; ROS, reactive oxygen species; S100β, S100 calcium-binding protein β; TNF-α, tumor necrosis factor-α .

The systemic inflammatory response triggered by surgical trauma constitutes a central pathological mechanism of postoperative delirium (POD), particularly following major procedures such as cardiac surgery. Systemic inflammation can compromise the blood-brain barrier (BBB) and activate microglial inflammatory responses (Figure 1) Surgical trauma activates neuroinflammation through multiple pathways, ultimately leading to acute brain dysfunction. Disruption of the blood-brain barrier (BBB) plays a critical role in this process. Studies have demonstrated that the cerebrospinal fluid/plasma albumin ratio (CPAR) and plasma levels of S100B are significantly elevated in patients with delirium, with the degree of increase positively correlating with delirium severity (Taylor et al., 2022). These abnormalities indicate increased BBB permeability, facilitating the invasion of peripheral inflammatory mediators into the central nervous system. Intraoperative blood loss and hypotension are significant predictors of BBB damage, promoting plasminogen activation and exacerbating the inflammatory cascade and vascular endothelial injury (Taylor et al., 2022). Furthermore, perioperative increases in cerebrospinal fluid (CSF) concentrations of fibrinogen have been shown to correlate with markers of BBB disruption, inflammation, and neuronal dysfunction (Payne et al., 2024).

Inadequate postoperative pain control may also be an important contributing factor to delirium. From a biological perspective, pain and delirium may be linked via shared and overlapping mechanisms (Devlin et al., 2018). Enhanced responsiveness to pain stimuli is regulated by the release of chemokines, cytokines, and glutamate, which can concurrently promote BBB breakdown (Devlin et al., 2018). Compelling evidence for perioperative neuronal injury comes from a large cohort study analyzing CSF samples (Watne et al., 2023). This study associated neuroinflammation with elevated levels of the neurotoxic metabolite quinolinic acid, resulting from activation of indoleamine 2,3-dioxygenase in the kynurenine pathway. Quinolinic acid activates NMDA-type glutamate receptors, inducing excitotoxicity, while simultaneously suppressing glycolysis. Notably, CSF levels of quinolinic acid were associated with delirium, increased neurofilament light chain (NfL), and survival outcomes in both surgical and medical patients (Watne et al., 2023). These findings provide a crucial link between inflammation and neuronal injury in the pathogenesis of delirium.

Various experimental models of postoperative cognitive impairment implicate the NOD-, LRR- and pyrin domain-containing protein 3 (NLRP3) inflammasome pathway in delirium. Inhibition of this inflammatory pathway and its effector, caspase-1, reduces the expression of inflammatory biomarkers and mitigates perioperative cognitive deficits (Li et al., 2023; Zhang et al., 2022). In summary, surgery-induced neuroinflammation promotes the development of postoperative delirium through a convergent mechanism involving BBB disruption, generation of neurotoxic metabolites, and activation of specific inflammasome pathways.

## Frailty and delirium in late life: two faces of age-driven neuroinflammation and brain-reserve depletion

### Neuroinflammation and cerebral reserve depletion

The risk of developing delirium is markedly elevated in the elderly population, with over 50% of hospitalized patients aged 65 years and older experiencing this complication (Inouye et al., 2014). This heightened susceptibility is fundamentally linked to age-associated alterations in neuroinflammatory regulation and diminished cerebral resilience.

### Core mechanism: primed neuroinflammation in the aging brain

The aged brain exists in a state of chronic, low-grade inflammation, characterized by baseline microglial activation and elevated pro-inflammatory cytokine levels. Upon exposure to an acute physiological stressor such as surgery or infection, this primed state precipitates an exaggerated and dysregulated neuroinflammatory response. Preclinical evidence underscores this phenomenon: following lipopolysaccharide (LPS) challenge, aged mice exhibit a threefold greater increase in hippocampal IL-1β expression compared to young controls, which correlates with significant cognitive deficits. This maladaptive response is driven by a microglial phenotypic shift; transcriptomic analyses reveal that aged microglia constitutively express high levels of pattern recognition receptors like Clec7a and demonstrate hyper-responsiveness to inflammatory stimuli (Healy et al., 2024).

### Neural connectivity: a dimension of compromised resilience

Beyond molecular inflammation, the aging brain exhibits vulnerabilities in functional neural networks. Research indicates that delirium is associated with impaired feedback connectivity from the left superior temporal gyrus (l-STG) to the auditory cortex. The strength of this connection is negatively correlated with both delirium severity and the magnitude of systemic inflammation (Gjini et al., 2024), suggesting that disrupted neural communication represents another critical facet of the brain’s diminished capacity to manage stress.

### Frailty and delirium: converging pathways of vulnerability

Advanced age, frailty, and a pro-inflammatory state are deeply interlinked. Systemic inflammation, driven by factors such as chronic diseases (e.g., cardiovascular disease, diabetes), sedentariness, malnutrition, and psychosocial stress, promotes frailty through pathways including sarcopenia, insulin resistance, and endothelial dysfunction (Mosharaf et al., 2025). In contrast, delirium is typically triggered by an acute inflammatory insult (e.g., infection, surgery) that leads to a surge of pro-inflammatory cytokines, which then disrupt central nervous system homeostasis (Vetrano et al., 2021).

### A unifying hypothesis: two sides of the same coin

A convergent framework posits that both frailty and delirium are clinical expressions of a common underlying biological vulnerability. This vulnerability stems from the age-progressive depletion of physiological reserves and compensatory mechanisms. Frailty serves as a robust clinical proxy for this accumulated deficit burden (Gonçalves et al., 2022), representing a state of reduced systemic resilience where minor stressors can precipitate functional decline. Analogously, delirium likely signifies a failure of cerebral adaptive capacity—an acute brain dysfunction that emerges when the brain’s functional reserves are insufficient to meet the metabolic and inflammatory demands imposed by a stressor (Bellelli et al., 2024). Thus, frailty and delirium can be viewed as two interrelated faces of the same process: age-driven erosion of homeostasis and reserve across systemic and cerebral domains.

Although infection, surgery, trauma, hypoxia, and frailty differ clinically, they may promote delirium through overlapping biological pathways. Peripheral inflammation activates circulating cytokines, complement proteins, endothelial adhesion molecules, extracellular vesicles, and damage-associated molecular patterns. These signals can affect the brain through humoral routes, neural afferents, endothelial activation, and disruption of the blood–brain barrier. Within the central nervous system, inflammatory signaling promotes microglial activation, astrocytic dysfunction, oxidative stress, mitochondrial injury, impaired synaptic plasticity, and altered neurotransmission. Aging and frailty may further amplify this response through microglial priming and reduced cognitive reserve. Therefore, delirium in critical illness is unlikely to reflect a single pathway; rather, it emerges from the convergence of systemic inflammation with impaired vascular, metabolic, glial, and neuronal homeostasis.

## Inflammatory markers and the prediction of delirium

The major categories of clinical evidence linking inflammation and delirium are summarized in Table 1, and candidate biomarkers for inflammation-associated delirium in critically ill patients are listed in Table 2. Inflammatory and neurodegenerative biomarkers jointly forecast delirium, yet they operate through distinct mechanistic strata. Two large-scale meta-analyses that aggregated >40 studies identified nine serum proteins—IL-6, CRP, IL-8, IL-10, MCP-1, GFAP, IL-1β, neurofilament light (NfL), and the neurotrophic factor IGF-1—as being reproducibly altered in post-operative delirium (POD). In parallel, a multicenter prospective cohort (*n* = 1,043) demonstrated that elevated IL-6, IL-8, IL-10, TNF-α, and TNFR1, coupled with reduced protein C, independently predicted delirium or coma within 24 h after measurement (Brummel et al., 2024). Nevertheless, 1-year neurocognitive follow-up of the same ICU cohort revealed that cumulative delirium duration predicted persistent self-reported cognitive impairment without significant interaction with the aforementioned inflammatory panel, indicating that downstream neurodegenerative processes—rather than sustained systemic inflammation—maintain long-term deficits (Wolters et al., 2017).

**TABLE 1:** Summary of major clinical evidence linking inflammation and delirium.

Evidence category	Representative study types	Main findings	Strengths	Key limitations	Interpretation for clinical practice
Mechanistic association	Observational cohort studies (prospective and retrospective)	Positive correlation between elevated levels of inflammatory mediators (e.g., IL-6, CRP, TNF-α) and the incidence, severity, or duration of delirium (Brummel et al., 2024; Mosharaf et al., 2025).	Demonstrates biological plausibility; identifies potential pathways (e.g., BBB disruption, neuroinflammation).	Heterogeneity in delirium assessment tools, patient populations (ICU vs. surgical), and confounding factors (e.g., sedation).	Inflammation is a critical, though multifactorial, pathophysiological hub for delirium development in the critically ill.
Preclinical models	Animal studies (sepsis, surgery, aging models)	Induction of systemic inflammation triggers microglial priming/activation, neurovascular unit dysfunction, and cognitive/behavioral deficits (Chung et al., 2023; Heneka et al., 2015).	Allows detailed tissue-level analysis of cellular mechanisms and temporal dynamics (e.g., NLRP3 activation).	Translation of animal “delirium-like behaviors” to clinical human delirium is difficult and requires caution.	Preclinical data strongly support a causal role for neurovascular and glial dysfunction driven by peripheral inflammation.
hTherapeutic and prevention trials	Clinical trials (pharmacological agents, e.g., dexmedetomidine, statins)	Some agents show potential to reduce delirium incidence or duration (e.g., dexmedetomidine), but many have mixed, conflicting, or negative results (Skrobik et al., 2018; Page et al., 2024; Shehabi et al., 2019).	Provides evidence from controlled interventions regarding potential clinical benefits.	Limited focus on long-term cognitive outcomes; conflicting evidence across different agents and populations.	Current evidence does not support a “simple anti-inflammatory pharmacological solution”; management relies on non-pharmacological bundles.

**TABLE 2:** Candidate biomarkers for inflammation-associated delirium in critically ill patients.

Biomarker	Main biological source	Pathophysiological interpretation	Timing/dynamics	Strengths	Limitations/ confounders	Potential clinical utility	Current translational readiness
IL-6 (Interleukin-6)	Immune cells (macrophages, monocytes), endothelial cells	Systemic cytokine activation; early inflammatory signal	Early, rapid rise following acute insult (e.g., sepsis, surgery)	Sensitive early inflammatory signal; associated with sepsis severity	Low specificity for delirium; strongly influenced by infection, surgery, and trauma	Risk stratification and identifying patients with high inflammatory burden	Experimental/research
CRP (C-reactive protein)	Liver (in response to IL-6)	Acute-phase response; systemic inflammatory marker	Delayed kinetics compared to cytokines (rises within 24–48 h)	Widely available and inexpensive in clinical practice	Non-specific; reflects generalized inflammation; delayed rise limits early prediction	Adjunctive systemic inflammatory marker; potentially for longitudinal monitoring	Clinical/research
S100β	Astrocytes	Astroglial injury; possible blood-brain barrier (BBB) disruption	Acute phase; relatively short half-life	May reflect acute brain injury or BBB permeability changes	Extracranial sources (e.g., adipose tissue, bone); affected by trauma; assay variability	Experimental/ adjunctive marker of glial activation or BBB dysfunction	Experimental/ research
GFAP (Glial fibrillary acidic protein)	Astrocytes (cytoskeleton)	Astroglial injury or activation	Early detection; may rise rapidly after CNS insult	More brain-enriched than CRP or IL-6; promising early detection marker	Timing-dependent release; limited validation specifically in diverse ICU cohorts	Promising early detection marker of glial injury/activation	Promising/research
Tau	Neurons (axons)	Neuronal and axonal injury; link to neurodegeneration	May rise later or show sustained elevation after acute insult	Directly reflects neuronal injury; strong biological plausibility for long-term impairment	Not delirium-specific; kinetics may limit early prediction; affected by age/comorbidities	Potential for long-term risk stratification and mechanistic studies	Experimental/research
NfL (Neurofilament light chain)	Neurons (axons)	Neuroaxonal injury; marker of structural damage	More sustained elevation, often peaking days to weeks later	Sensitive marker of neuroaxonal injury; associated with chronic neurological disease	Influenced by age, chronic neurological conditions, and renal dysfunction; slow kinetics	Long-term risk stratification and post-ICU cognitive trajectory prediction	Promising/research

Among emerging biomarkers, astrocytic injury signatures offer complementary temporal resolution. Plasma S100β independently predicted incident delirium and delirium-related mortality by reflecting blood–brain barrier disruption (Khan et al., 2020), whereas GFAP elevations preceded clinical manifestation of hypoactive delirium by approximately 12 h and identified subsyndromal states with 82% sensitivity (Liu et al., 2023). Total tau, a marker of axonal injury, rose >15 pg/mL on post-operative day 1 and predicted delirium with 91% sensitivity and 79% specificity; its concentration correlated more strongly with delirium severity than IL-6, underscoring its utility as a direct index of ongoing neurodegeneration (Ballweg et al., 2021; Yao et al., 2025). Finally, readily accessible hematological indices—the red-cell distribution width-to-albumin ratio (RDW/Alb) and the systemic immune-inflammation index (SII)—provided low-cost, real-time risk stratification for sepsis-associated and non-cardiac post-operative delirium, respectively (Yao et al., 2025; Song et al., 2022), while NfL remains a promising but incompletely validated marker of delirium-related neuroaxonal injury (Krogseth et al., 2023).

Collectively,these data support a hierarchical biomarker paradigm in which acute inflammatory triggers (IL-6, CRP, TNF-α), astrocytic and neuronal injury signatures (S100β, GFAP, tau), and systemic vulnerability metrics (RDW/Alb, SII) converge to predict both the onset and the enduring cognitive sequelae of delirium.

Delirium in critical illness is associated with persistent cognitive impairment after hospital discharge, but the biological relationship between acute delirium and chronic neurodegeneration remains incompletely understood. Systemic inflammation may trigger sustained microglial activation, blood–brain barrier dysfunction, oxidative stress, and mitochondrial injury, thereby impairing synaptic plasticity and neuronal repair. Biomarkers such as tau and NfL suggest that some patients with delirium may experience neuronal and axonal injury during acute illness. In vulnerable individuals, including older adults and patients with pre-existing cognitive impairment, acute inflammatory insults may accelerate tau phosphorylation, amyloid-related pathways, or other neurodegenerative processes. However, delirium may also function as a marker of reduced cognitive reserve rather than a direct cause of neurodegeneration. Longitudinal studies combining inflammatory biomarkers, neuronal injury markers, neuroimaging, and post-discharge cognitive testing are needed to clarify whether anti-inflammatory strategies can reduce long-term cognitive decline.

Although inflammatory and neuronal injury biomarkers are biologically plausible indicators of delirium risk, none currently has sufficient specificity to function as a stand-alone diagnostic test. IL-6 and CRP are readily available and reflect systemic inflammatory burden, but they are strongly influenced by infection severity, surgery, tissue injury, and organ dysfunction. S100β and GFAP may provide information about astrocytic activation or blood–brain barrier injury, but their interpretation is complicated by extracranial sources, trauma, assay variability, and timing of release. Tau and NfL are more closely associated with neuronal and axonal injury, but their kinetics may limit their utility for early delirium prediction. Future clinical application will likely require multimarker panels integrated with clinical variables such as age, frailty, illness severity, sedative exposure, and delirium screening results.

The current evidence base is heterogeneous. Studies differ substantially in patient population, delirium definition, assessment instrument, timing of biomarker sampling, severity of illness, sedative and analgesic exposure, and adjustment for confounding variables. Many biomarker studies are observational and therefore cannot determine whether inflammatory markers are causal mediators of delirium, correlates of critical illness severity, or indicators of underlying brain vulnerability. Consequently, inflammatory and neuronal injury biomarkers should currently be interpreted as adjunctive risk indicators rather than stand-alone diagnostic tools.

## Controlling the inflammatory response for the therapeutic efficacy of delirium

### Current situation of treating delirium

Anti-inflammatory and mechanism-targeted interventions for delirium in critically ill patients are detailed in Table 3. Although the prevalence of delirium is high and is associated with serious adverse outcomes, the underlying pathological mechanisms of delirium remain poorly understood. Current management strategies focus on supportive care, including addressing the root causes (such as infection, metabolic imbalance, and drug use), optimizing environmental factors, and adopting non-pharmacological interventions such as cognitive rehabilitation and sleep management (Wilson et al., 2020). However, these methods often yield limited benefits, especially in the elderly and critically ill patients. Drug treatment also faces challenges, as there is no conclusive evidence supporting the effectiveness of cholinesterase inhibitors, antipsychotics, melatonin, or dexmedetomidine in preventing delirium.

**TABLE 3:** Anti-inflammatory and mechanism-targeted interventions for delirium in critically ill patients.

Intervention	Proposed mechanism	Current evidence	Main supportive findings	Negative/ conflicting findings	Safety concerns	Clinical readiness
Non-pharmacological ICU care bundles (e.g., ABCDEF)	Multifactorial reduction of noxious stimuli, sleep optimization, mobilization	Strong supportive evidence	Consistently associated with reduced delirium incidence, duration, and improved outcomes	Effectiveness depends on high fidelity implementation	No major safety concerns	Established clinical standard
Dexmedetomidine	Sympatholysis, sedation, analgesia-sparing; indirect anti-inflammatory modulation	Supportive in specific contexts	Reduced delirium vs. benzodiazepine-based sedation	Benefit may reflect lighter sedation rather than anti-inflammatory effects	Bradycardia, hypotension	Clinically available
NSAIDs/COX inhibitors	Inhibition of prostaglandin synthesis; reduction of inflammation	Mixed/Weak evidence	Potential benefit in specific surgical settings	Not consistently effective in general ICU; risk-benefit concerns	GI bleeding, renal dysfunction, CV risks	Clinical availability, weak evidence
Statins	Pleiotropic anti-inflammatory and endothelial-protective effects	Mixed/ limited evidence	Some observational data link to reduced delirium	Failed to show benefit in large RCTs	Myopathy, liver enzyme elevation	Available, no routine use support
Melatonin/ ramelteon	Sleep-wake regulation; potential antioxidant/ anti-inflammatory	Mixed/ conflicting evidence	Potential in sleep-deprived ICU or postoperative patients	RCT results inconsistent for delirium prevention	Generally well-tolerated; potential sedation	Available, weak routine use evidence
Antipsychotics	Dopamine and serotonin receptor antagonism; symptom control	Weak/conflicting evidence	May be used for severe agitation or psychotic symptoms	Do not consistently reduce delirium duration, severity, or mortality	QT prolongation, extrapyramidal symptoms, mortality risk	Reserved for symptom management
Cholinesterase inhibitors (e.g., rivastigmine)	Augmentation of cholinergic neurotransmission	Negative evidence	No significant findings for delirium prevention/treatment	Large RCTs showed no benefit; potentially increased mortality	GI side effects, bradycardia	Not for delirium prevention/ treatment
GLP-1 receptor agonists	Anti-inflammatory and neuroprotective effects	Preliminary/ emerging (preclinical)	Strong preclinical neuroprotective effects	Lacks large-scale clinical validation	GI side effects, hypoglycemia	Investigational; needs trials
Cytokine-targeted therapies (e.g., anti-IL-6)	Direct inhibition of systemic cytokine signaling	Very preliminary/ Emerging	Potential in hyper-inflammatory states (sepsis/COVID-19)	Lacks target validation for delirium	Immunosuppression, infection risk	Investigational; needs targeted trials
Neuromodulation (e.g., tDCS, VNS)	Neural network modulation; potential anti-inflammatory effects	Preliminary/ emerging	Some positive results in small studies or surgical cohorts	Lacks robust validation from large RCTs	Skin irritation (tDCS), device complications (VNS)	Investigational; needs validation

### Relationship between drugs controlling inflammation levels and delirium

#### Hormone bundle therapy

The role of hormones in the management of patients with delirium is not well defined. A perioperative anti-inflammatory bundle combining dexmedetomidine, glucocorticoids, ulinastatin, and nonsteroidal anti-inflammatory drugs reduced the risk for postoperative delirium in older patients undergoing hip fracture surgery in a study of 132 patients. Systemic inflammation mediated the protective effect of this intervention (Nawan et al., 2025). An article has suggested that high-dose methylprednisolone prophylactic treatment can alleviate the systemic inflammatory response during open-heart surgery under cardiopulmonary bypass, but it cannot prevent or reduce the increase in blood-brain barrier permeability or neuroinflammatory response (Danielson et al., 2018). A study in mice suggests caffeine as a potential delirium therapy (Sultan et al., 2021), with secondary analysis of trial data suggesting a potential benefit on postanaesthesia care unit delirium.

#### Relationship of delirium-control medications with inflammation

**FIGURE 2:**
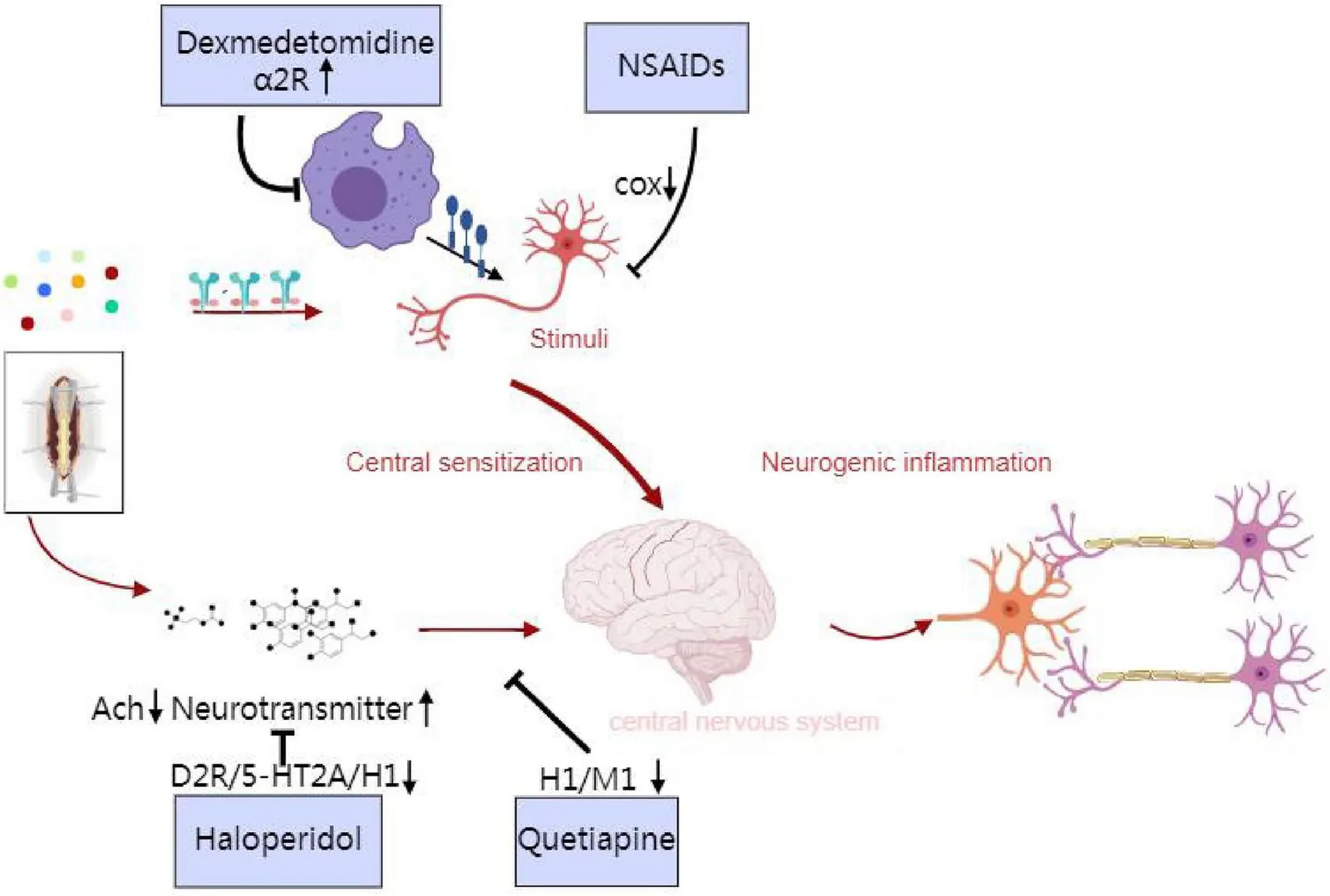
Drug treatment for delirium and inflammatory pathways. Solid arrows indicate mechanisms supported by clinical or experimental evidence. Black solid arrows represent direct inflammatory or pathophysiological pathways (e.g., cytokine release, cellular activation). Red solid arrows indicate inhibitory pharmacological blockade or suppression of target pathways (e.g., anti-inflammatory drug action). Truncated line segments (T-bar or flat-head terminations) denote inhibitory or blocking effects at the endpoint. Dashed arrows indicate proposed or indirect pathways requiring further validation. BBB, blood-brain barrier; CAIP, cholinergic anti-inflammatory pathway; COX, cyclooxygenase; CNS, central nervous system; DAMPs, damage-associated molecular patterns; EV, extracellular vesicle; GDF15, growth differentiation factor 15; GLP-1RA, glucagon-like peptide-1 receptor agonist; IL, interleukin; NLRP3, NOD-like receptor family pyrin domain-containing 3; NSAIDs, non-steroidal anti-inflammatory drugs; SGLT2-i, sodium-glucose cotransporter 2 inhibitor; VNS, vagus nerve stimulation.

Surgical trauma triggers proinflammatory cascades and neurotransmitter imbalances, key contributors to postoperative delirium (POD). Pharmacological agents targeting these pathways show promise in POD prevention and management. Furthermore, targeted pharmacological blockade, such as the use of NSAIDs or GLP-1RAs, can inhibit these neuroinflammatory pathways (Figure 2).

Dexmedetomidine, a nonspecific α2-adrenergic receptor agonist, exerts antiinflammatory effects that counteract surgery-induced proinflammatory mechanisms. Furthermore, it promotes cognitive preservation by mimicking natural sleep pathways and reducing opioid requirements, although its long-term effects warrant further investigation (Strada et al., 2024). Similarly, evidence suggests that perioperative NSAIDs (Non-Steroidal Anti-Inflammatory Drugs) are associated with a reduced incidence of POD, likely attributable to their antiinflammatory properties mitigating surgery-induced neuroinflammation (Li et al., 2021).

Neurotransmitter dysregulation is another critical factor. Haloperidol possesses potent antiadrenergic properties, along with mild antihistaminic, antiserotonergic, and peripheral anticholinergic activities, counteracting multiple pathways implicated in delirium development (Zhao et al., 2025). Quetiapine, commonly used for ICU delirium, acts primarily as a sedative at lower doses by blocking histamine H1 and muscarinic receptors, promoting sleep and reducing anxiety while minimizing delirium exacerbation risk. Its rapid absorption (peak plasma levels within 1 h), major half-life of 7 h, and active metabolite (N-dialkylquetiapine, half-life ∼12 h) contribute to sustained symptom control. Notably, quetiapine is metabolized hepatically via CYP3A4, making its efficacy and dosing highly dependent on liver function (Menozzi et al., 2025).

The neuroimmune interaction underlying these effects can be conceptualized through the cholinergic anti-inflammatory pathway (CAIP). The CAIP describes a physiological mechanism where the central nervous system (CNS) modulates systemic inflammation via vagus nerve signaling. This pathway represents a potential therapeutic target for mitigating neuroinflammation associated with conditions like POD (Han et al., 2017).

#### Novel therapeutics targeting inflammatory pathways under investigation or in development for delirium

Evidence from experimental and clinical studies suggests that several pharmacological classes may confer protection against delirium by modulating neuroinflammation, synaptic integrity, and blood-brain barrier (BBB) function. The following summarizes key interventions according to their primary mechanisms of action:

(1) **Antidiabetic Agents**
  Ishibashi et al. (2024) proposed that SGLT2 inhibitors (SGLT2-i), GLP-1 receptor agonists (GLP-1RAs), and DPP-4 inhibitors (DPP4-i) may exert protective effects against delirium episodes. Supporting evidence includes:
  *GLP-1RAs* (e.g., liraglutide): Experimental models show that liraglutide inhibits NLRP3 inflammasome activation and microglial dysfunction, while counteracting surgery-induced synaptic loss and plasticity impairment. In aged mice after cardiac surgery, liraglutide attenuated delirium-like behaviors, likely through enhancing microglial mitophagy, reducing neuroinflammation, and preserving synaptic integrity (Jia et al., 2024).
  *SGLT2 inhibitors*: These agents have been shown to improve BBB function and enhance the activity of astrocytes, microglia, and oligodendrocytes, potentially contributing to improved cognitive outcomes (Pawlos et al., 2021).
(2) **Targeting neuroinflammation and immune cell trafficking**
  *Inhibition of inflammatory monocyte recruitment*: Preventing the migration of inflammatory monocytes across the BBB represents a promising therapeutic strategy. Preclinical models link this intervention to reduced neuroinflammation and cognitive impairment (Andonegui et al., 2018).
(3) **Statins**
  Exploratory studies suggest that statins may protect against delirium by modifying endothelial cell function and reducing inflammation. This is supported by a prospective cohort study showing that statin use in critically ill patients was associated with a lower incidence of delirium (Morandi et al., 2011).
(4) **Modulation of oxidative stress and inflammation**
  *Niacinamide (Vitamin B3)*: An animal study found that surgery increased oxidation of ryanodine receptor calcium channels and decreased BDNF levels. Niacinamide alleviated the postoperative inflammatory response and prevented cognitive decline and synaptic plasticity defects, likely through reducing IL-1β and CD38 protein levels (Guncay et al., 2025).
(5) **Neuromodulation**
  *Vagus nerve stimulation (VNS):* A prospective study demonstrated that VNS can reduce inflammation, alleviate postoperative pain, and promote recovery, likely mediated by enhanced acetylcholine release and activation of the cholinergic anti-inflammatory pathway (Zhang et al., 2025)
(6) **Cytokine-targeted therapy**
  *Anti-GDF15 antibody:* Studies in septic mice revealed increased brain levels of growth differentiation factor 15 (GDF15). Targeting GDF15 with a neutralizing antibody improved sepsis-induced cognitive and memory impairment by attenuating microglial inflammatory responses and phagocytic activity, suggesting GDF15 as a potential therapeutic target for sepsis-associated delirium (Chen et al., 2025).
(7) **Specific anti-inflammatory targets**
  *IL-1 blockers:* Agents designed to inhibit interleukin-1 signaling pathways may mitigate neuroinflammation (Gust et al., 2020).
  *CSF1R inhibitors* (e.g., PLX5622): These compounds inhibit the colony-stimulating factor 1 receptor and are under preclinical investigation for their ability to deplete activated microglia and reduce neuroinflammation (Tarale and Alam, 2022).

Current evidence does not support a simple anti-inflammatory pharmacological solution for delirium prevention or treatment. Non-pharmacological multicomponent prevention, minimization of deliriogenic medications, early mobilization, sleep promotion, pain control, and light sedation remain the foundation of delirium management. Pharmacological agents may be considered in selected contexts, but most have not shown consistent benefit across heterogeneous ICU populations. Dexmedetomidine may reduce delirium compared with benzodiazepine-based sedation in some mechanically ventilated patients, yet its effects may reflect lighter sedation, analgesia-sparing properties, sleep modulation, and sympatholysis rather than direct anti-inflammatory activity alone. Antipsychotics have not consistently reduced delirium duration or mortality and should generally be reserved for severe agitation or distressing symptoms. Emerging approaches, including GLP-1 receptor agonists, cytokine-targeted therapies, and neuromodulation, remain investigational and require well-designed clinical trials.

In summary, these interventions may mitigate delirium risk or pathology through multiple convergent mechanisms, including suppression of neuroinflammation (e.g., via NLRP3, microglia, or GDF15), enhancement of BBB integrity, promotion of synaptic plasticity, modulation of oxidative stress, and activation of anti-inflammatory neural circuits. Future clinical research should prioritize well-designed trials to evaluate the efficacy of these promising pharmacological and neuromodulatory approaches in preventing or treating delirium in relevant patient populations.

## Future directions

Future research should prioritize large-scale, multi-center longitudinal cohorts that integrate a wider array of inflammatory biomarkers—including proteomic and metabolomic profiling—with robust, standardized diagnostic criteria for delirium and rigorous confounder adjustment. Exploring temporal interactions between biomarkers, their cumulative effects, and metabolic factors may yield more comprehensive mechanistic models and reveal novel therapeutic targets. Precision medicine trials targeting specific inflammatory endotypes (e.g., hyper-inflammatory versus immunoparalyzed states), coupled with the development and prospective validation of multimarker panels integrated with clinical risk scores, hold promise for individualized delirium prevention and improved real-world diagnostic utility.

## Conclusion

The evidence synthesized in this review positions systemic inflammation as a cornerstone of delirium pathogenesis in critical illness. Triggers such as infection, surgery, and frailty converge on shared neuroinflammatory pathways, driving acute brain dysfunction. This cascade is reflected by a distinct profile of biomarkers, from inflammatory cytokines to indicators of neural injury, enabling improved prediction of delirium risk and outcomes. Moving beyond supportive care, modulating this inflammatory response represents the most promising therapeutic frontier. Validating these biomarkers and testing novel anti-inflammatory agents in rigorous clinical trials are now critical next steps to mitigate the daunting burden of delirium.

## Data Availability

The original contributions presented in this study are included in the article/supplementary material, further inquiries can be directed to the corresponding author.
